# Associations between Glasgow Coma Scale trajectories and 28-day survival rate in patients with sepsis-associated encephalopathy: insights from longitudinal group trajectory modeling

**DOI:** 10.3389/fneur.2025.1607946

**Published:** 2025-09-02

**Authors:** Dayong Shen, Su Zhou, Han Wu, Wei Zhang, Zeheng Li, Jie Sun

**Affiliations:** ^1^Department of Neurology, The Affiliated Hospital of Xuzhou Medical University, Xuzhou, Jiangsu, China; ^2^Xuzhou Central Hospital, Xuzhou, Jiangsu, China; ^3^Soochow University, Suzhou, Jiangsu Province, China; ^4^Xuzhou Clinical College Affiliated to Xuzhou Medical University, Xuzhou, Jiangsu, China

**Keywords:** sepsis-associated encephalopathy, Glasgow Coma Scale, prognosis, group-based trajectory modeling, survival rate

## Abstract

**Background:**

Sepsis-associated encephalopathy (SAE) is a common and serious complication of sepsis, characterized by altered consciousness and cognitive dysfunction. This study aimed to investigate the relationship between dynamic changes in Glasgow Coma Scale (GCS) scores during the 5 days prior to ICU admission and 28-day survive rate in patients with SAE.

**Patients and methods:**

A retrospective cohort study was conducted using data extracted from the MIMIC-IV v2.0 database. Patients diagnosed with SAE and having daily GCS measurements for 5 days prior to ICU admission were included. A total of 76,943 intensive care unit (ICU) admissions were screened, and 1,389 patients were finally analyzed. Group-based trajectory modeling (GBTM) was employed to categorize patients based on their GCS trajectories, and survival analysis was conducted using Kaplan–Meier curves and Cox regression models.

**Results:**

Four distinct GCS trajectory classes were identified: Class 1 (rapid increase), Class 2 (slow increase), Class 3 (rapid decrease), and Class 4 (persistent high level). Patients in Class 4, who had persistent high GCS scores, exhibited the highest survival rate of 82.4 ± 1.2%. In contrast, Class 1, the rapid increase group, had a survival rate of 74.1 ± 3.2%. Both Class 2 (slow increase) and Class 3 (rapid decrease) groups had lower survival rates, with Class 2 at 65.7 ± 5.7% and Class 3 at 65.2 ± 3.6% (*p* < 0.001). Multivariate Cox regression analysis revealed that patients in classes 1, class 2, and class3 had significantly increased mortality risk compared to Class 4 (*p* < 0.05). Subgroup analyses indicated that the effect of GCS trajectory on 28-day survive rate was consistent across different subgroups of patients. The ICU stay duration among the four groups of patients showed no statistical difference (*p* = 0.291).

**Conclusion:**

The dynamic changes in GCS scores over the 5 days prior to ICU admission was significantly associated with 28-day survive rate in patients with septic encephalopathy. Monitoring GCS fluctuations can provide valuable prognostic information in this population.

## 1 Background

Sepsis-associated encephalopathy (SAE) is a common and severe manifestation of sepsis, characterized by altered mental status ranging from mild confusion to deep coma (1). It occurs in up to 50% of patients with severe sepsis or septic shock and significantly contributes to the morbidity and mortality associated with these conditions (1, 2). The pathophysiology of septic encephalopathy is complex, involving a range of factors such as cytokine release, blood–brain barrier disruption, and altered neurotransmitter activity (3). Despite advances in the management of sepsis, SAE remains a major challenge in critical care medicine, both in terms of diagnosis and treatment. The Glasgow Coma Scale (GCS) score is widely used to assess the level of consciousness in critically ill patients, providing an objective measure of neurological function (4–6). However, most studies have focused on static GCS scores at the time of ICU admission as a predictor of mortality (6–8). Few studies have evaluated how changes in the GCS score over time, specifically its trajectory, may impact outcomes. This gap in knowledge is particularly important because cognitive fluctuations in sepsis-related encephalopathy often precede deterioration or recovery, and understanding these fluctuations could provide valuable prognostic information. Given that septic encephalopathy can evolve rapidly, the trajectory of GCS scores may offer critical insights into the patient’s prognosis. Previous studies have shown that early cognitive dysfunction in critically ill patients is associated with higher mortality, prolonged ICU stays, and long-term cognitive impairments (9–11). However, the relationship between the temporal changes in GCS scores and the risk of death specifically in patients with septic encephalopathy has not been well characterized.

Recent advances in statistical modeling, such as Group-Based Trajectory Modeling (GBTM), offer a promising method to analyze these temporal patterns and identify distinct subgroups of patients based on their clinical trajectories (12–14). GBTM has been widely used in other domains of critical care, such as predicting the trajectories of organ failure in septic patients, but its application to GCS trajectories in patients with SAE remains unexplored. This gap presents an opportunity to better understand the complex interplay between neurological function and sepsis progression.

Overall, the aim of this study was to explore the dynamic changes in GCS scores over time and their impact on 28-day survive in patients with SAE. By employing advanced statistical modeling techniques, we sought to identify distinct GCS trajectory groups and investigate how these trajectories were associated with patients’ short prognosis. Given the growing recognition of the importance of cognitive dysfunction in critically ill patients, this study provides valuable insights into how temporal assessments of neurological function can improve the prediction of prognosis and guide clinical decision-making in SAE.

## 2 Patients and methods

### 2.1 Study design and data source

This study utilized a retrospective cohort design aimed at analyzing the dynamic changes in GCS scores in patients with septic encephalopathy and their association with 28-day survive rate. The data for this study were obtained from the Medical Information Mart for Intensive Care database 2.0 (MIMIC-IV 2.0), an open-access and widely used clinical database that encompasses detailed medical records of adult patients treated in a large hospital intensive care unit ICU between 2008 and 2019. The MIMIC-IV database contains comprehensive information, including clinical variables, laboratory test results, treatment records, and patient outcome data, providing a robust foundation for this research.

### 2.2 Patient selection

**Figure 1:**
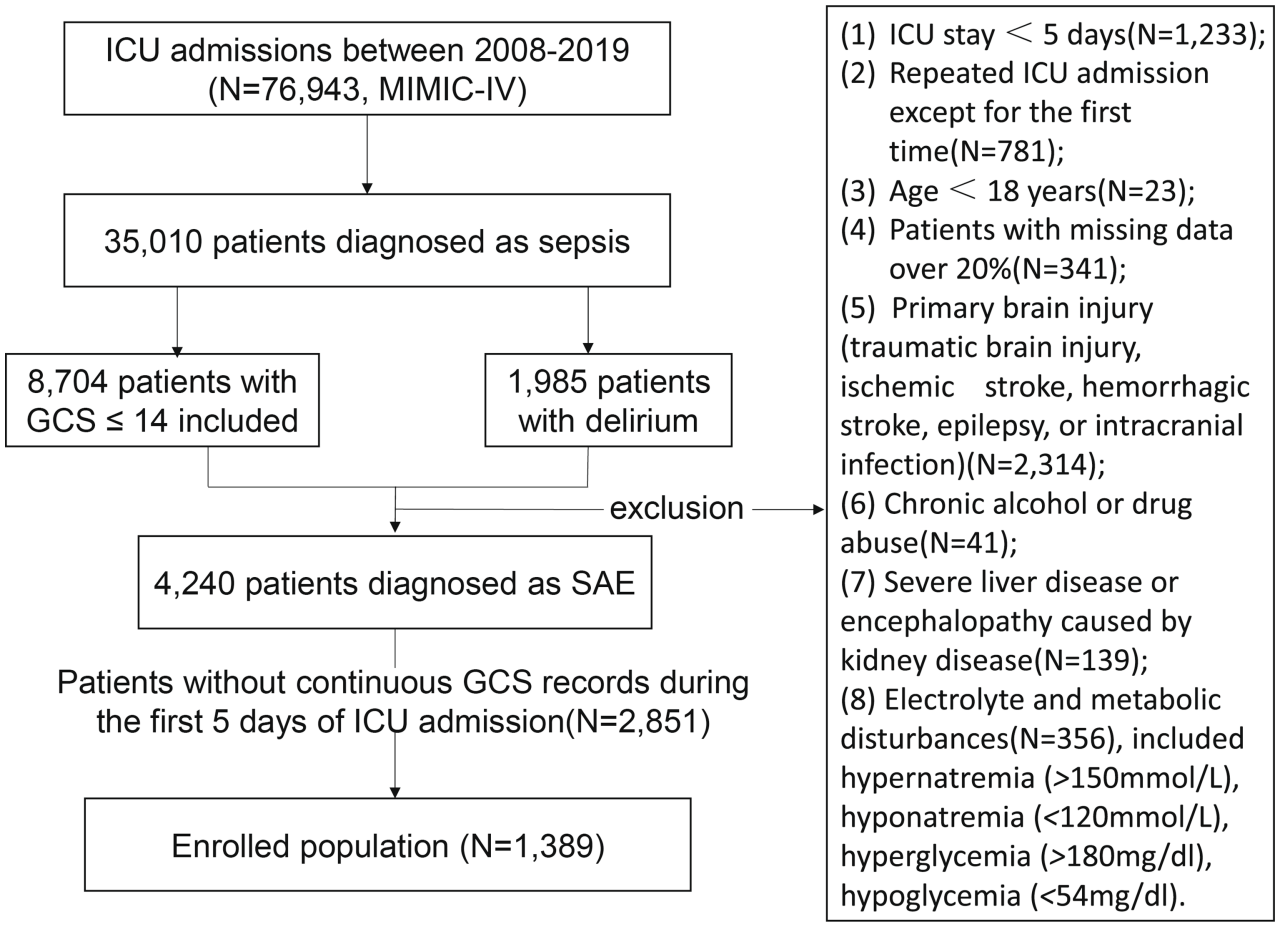
Screening process for patients with sepsis-associated encephalopathy.

Inclusion criteria for the study comprised: 1. patients aged 18 years or older, 2. Patients diagnosed as SAE were patients with sepsis who also exhibited cognitive dysfunction. The diagnosis of sepsis for patients was directly provided by the MIMIC database, based on the Sepsis 3.0 diagnostic criteria and the cognitive dysfunction was defined as a GCS score of 14 or lower or the presence of newly developed delirium (15). The following ICD codes were utilized for identifying sepsis patients in this study: 67020; 67,022; 67,024; 77,181; 99,591; 99,592; A021; A227; A267; A327; A40; A400; A401; A403; A408; A409; A41; A410; A4101; A4102; A411; A412; A413; A414; A415; A4150; A4151; A4152; A4153; A4159; A418; A4181; A4189; A419; A427; A5486; B377; O0337; O0387; O0487; O0737; O0882; O85; O8604; P36; P360; P361; P3610; P3619; P362; P363; P3630; P3639; P364; P365; P368; P369; R652; R6520; R6521; T8144; T8144XA; T8144XD; T8144XS; Y07436. These codes encompass a range of diagnoses related to sepsis and were essential for accurately identifying the cohort of patients included in the analysis. Exclusion criteria included: 1. patients with missing GCS data, 2. patients with ICU length of stay less than 120 h, 3. patients with a history of severe neurological diseases such as stroke or epilepsy prior to admission, and 4. patients with prior severe head trauma. Additionally, patients who lacked essential clinical data necessary for comprehensive analysis were excluded. Ultimately, 1,389 patients were included for statistical analysis in this study (Figure 1).

### 2.3 Data collection

Data extracted from the MIMIC-IV database included: basic demographic information such as age, gender, and race, clinical characteristics such as comorbidities, medication history prior to admission, and Sequential Organ Failure Assessment (SOFA) scores at ICU admission, and physiological parameters including heartrate, blood pressure, temperature, and clinical laboratory indicators (such as hemoglobin, platelet count, and white blood cell count), which are typically recorded within the first 24 h of ICU admission. Specifically, GCS scores were recorded on the day of ICU admission and throughout the following 4 days to capture fluctuations in the patients’ neurological status. The primary outcome measure for this study was 28-day survive rate after ICU admission. The secondary objective is to observe the ICU stay duration of four groups of patients.

### 2.4 GCS trajectory analysis

GBTM was employed to analyze the changes in GCS scores during the first 5 days following ICU admission ICU admission. GBTM is a grouping method that identifies distinct patterns of change. This analysis allowed researchers to categorize patients into four classes based on their GCS trajectories. The analytical steps included preprocessing the collected GCS data to ensure completeness, followed by the implementation of GBTM using statistical software such as R to determine the optimal number of groups and the quality of model fit. To determine the optimal number of trajectory classes, we utilized several criteria, including improvements in the absolute value of Bayesian Information Criterion (BIC) values, assignment probabilities greater than 70%, minimal class sizes exceeding 5% of the sample size, and our clinical experience.

### 2.5 Statistical analysis

Descriptive statistics were performed to summarize patient demographics and clinical variables. Categorical variables were described as numbers and percentages, and their differences among groups were compared using the Chi-squared test. Non-continuous variables and continuous variables that did not follow a normal distribution were described as median and interquartile range (IQR), and were analyzed using non-parametric methods (Mann–Whitney-Wilcoxon for two groups, Kruskal-Wallis for multiple groups). Continuous variables that followed a normal distribution were expressed as means and standard deviations, with Analysis of Variance (ANOVA) (for multiple groups) applied to these variables. Multivariable Cox proportional hazards regression analysis was conducted to evaluate the independent association between GCS trajectories and 28-day mortality, adjusting for potential confounding variables such as age, gender, and SOFA scores. Additionally, survival analysis was performed to compare survival rates among different groups, utilizing the log-rank test to compute survival curves. Statistical significance was set at *p* < 0.05 (two-tailed). All analyses were performed using R statistical software (version 4.3.0).

### 2.6 Ethical considerations

The study was approved by the Institutional Review Board (IRB) of the Massachusetts Institute of Technology (MIT) and the Beth Israel Deaconess Medical Center. Given that the data were de-identified, informed consent was waived.

## 3 Results

### 3.1 GCS trajectory analysis

**Figure 2:**
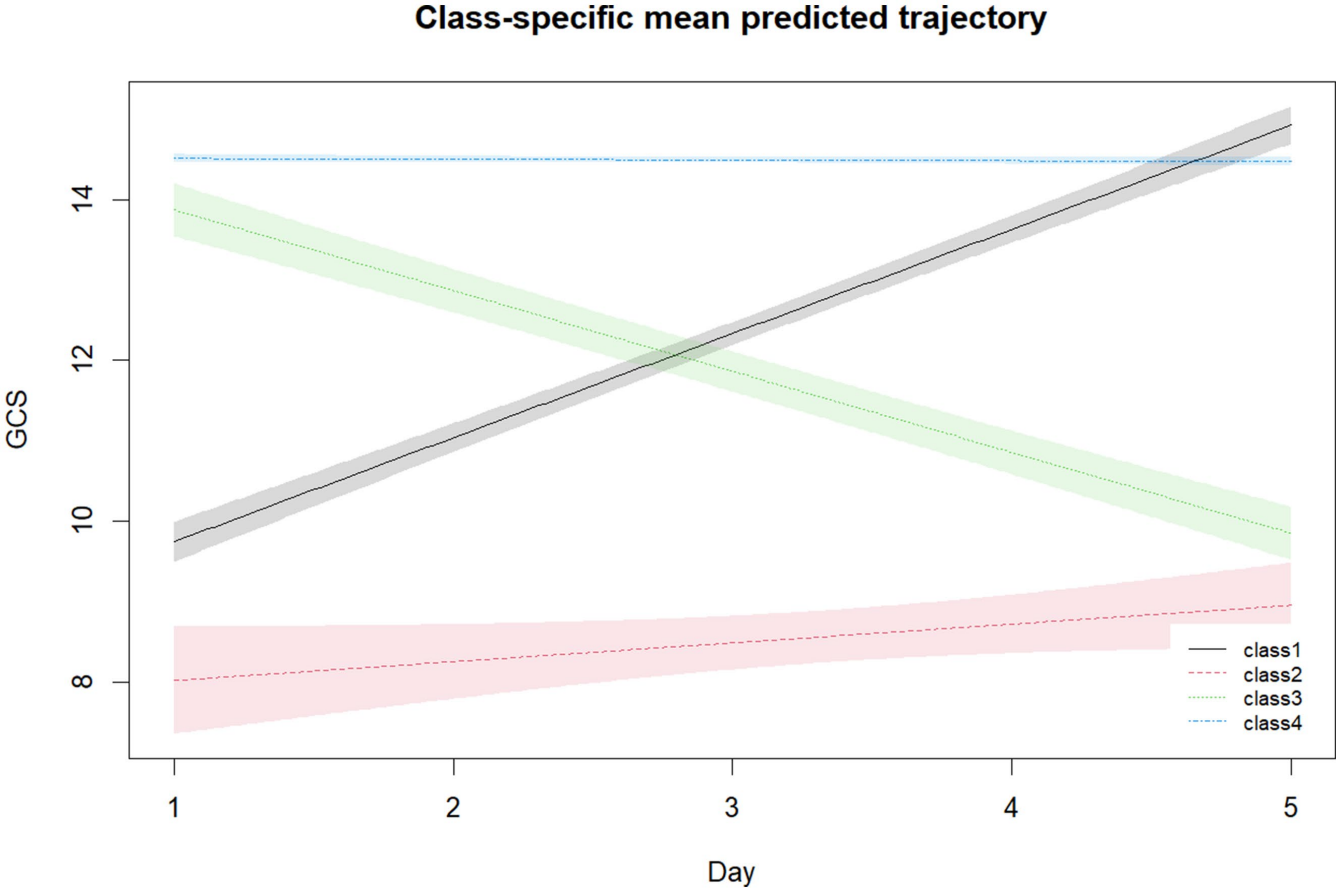
Trajectory model of GCS scores in enrolled patients. GCS, Glasgow Coma Scale.

A total of 1,389 patients diagnosed with septic encephalopathy were included in the final analysis. The mean age of the cohort was 63.7 ± 10.4 years, and 58.7% of the patients were male. The average SOFA score at ICU admission was 4.3 ± 3.6, indicating a high level of organ dysfunction among the patients. Using GBTM, four distinct GCS trajectory classes were identified based on the changes in GCS scores over the 5 days prior to ICU admission (Figure 2). Class 1, the rapid increase group, consisted of 185 patients (13.3%) who showed a significant improvement in their GCS scores, indicating a positive trend in consciousness. Class 2, the slow increase group, included 70 patients (5.1%) who experienced a more gradual improvement over time. Class 3, the rapid decrease group, represented 178 patients (12.8%), and these patients showed a sharp decline in GCS scores, suggesting deterioration in their mental status. The largest group, Class 4, which consisted of 956 patients (68.8%), showed relatively stable and high GCS scores throughout the monitoring period, reflecting a consistent level of consciousness. The average posterior probability for class 4 was 90.9% (Table 1). This confirms the reliability of the trajectory classifications and highlights the robustness of the model used.

**Table 1:** The Group-based trajectory modeling (GBTM) parameters for GCS trajectory grouping.

Number of classes	BIC	AvePP (%)	Class 1 (%)	Class 2 (%)	Class 3 (%)	Class 4 (%)	Class 5 (%)
1	54655.98	100	100				
2	51698.15	95.2	8.2	91.8			
3	50760.63	91.4	5.8	7.2	87.0		
4	50055.97	90.9	13.3	5.1	12.8	68.8	
5	50079.48	90.8	2.8	78.6	7.2	6.4	5.0

BIC, Bayesian Information Criteria; AvePP, Average Posterior Probabilities.

### 3.2 Baseline characteristics of the four trajectory classifications

When examining the baseline characteristics across the four GCS trajectory groups, significant differences were observed. Patients in the Class 1 trajectory group (rapid increase) were significantly younger, with a mean age of 59.4 ± 9.7 years, compared to those in the Class 4 group, whose mean age was 65.1 ± 11.2 years. In addition, the incidence of chronic pulmonary disease, myocardial infarction, and shock varied significantly between the groups (*p* < 0.05). These differences reflect the diverse clinical profiles of patients, which may influence both their GCS trajectories and survival outcomes. Baseline Characteristics of Patients in Four GCS Score Groups were described in Table 2.

**Table 2:** Baseline characteristics of patients in four GCS score groups.

Variable	Class 1 (*N* = 185, 13.3%)	Class 2 (*N* = 70, 5.1%)	Class 3 (*N* = 178, 12.8%)	Class 4 (*N* = 956, 68.8%)	*p-*value
Gender, *n* (%)					
Male	79(42.7)	35 (50.0)	99(55.6)	411 (43.0)	0.013
Female	106 (57.3)	35 (50.0)	79 (44.4)	545 (57.0)	
Myocardial infarction, *n* (%)					
No	158 (85.4)	67 (95.7)	155 (87.1)	832 (87.0)	0.163
Yes	27 (14.6)	3(4.3)	23(12.9)	124 (13.0)	
Congestive heart failure, *n* (%)					
No	138 (74.6)	47 (67.1)	136 (76.4)	686 (71.8)	0.385
Yes	47 (25.4)	23 (32.9)	42 (23.6)	270 (28.2)	
Chronic pulmonary disease, *n* (%)					
No	137 (74.1)	64 (91.4)	145 (81.5)	712 (74.5)	0.003
Yes	48 (25.9)	6 (8.6)	33 (18.5)	244 (25.5)	
Diabetes, *n* (%)					
No	132 (71.4)	50 (71.4)	135 (75.8)	737 (77.1)	0.308
Yes	53 (28.6)	20 (28.6)	43 (24.2)	219 (22.9)	
Renal disease, *n* (%)					
No	156 (84.3)	55 (78.6)	144 (80.9)	774 (81.0)	0.672
Yes	29 (15.7)	15 (21.4)	34 (19.1)	182 (19.0)	
Malignant cancer, *n* (%)					
No	163 (88.1)	59 (84.3)	160 (89.9)	839 (87.8)	0.672
Yes	22 (11.9)	11 (15.7)	18 (10.1)	117 (12.2)	
Peptic ulcer disease, *n* (%)					
No	181 (97.8)	69 (98.6)	174 (97.8)	927 (97.0)	0.763
Yes	4 (2.2)	1 (1.4)	4 (2.2)	29 (3.0)	
Mild liver disease, *n* (%)					
No	160 (86.5)	62 (88.6)	157 (88.2)	838 (87.7)	0.953
Yes	25 (13.5)	8 (11.4)	21 (11.8)	118 (12.3)	
Shock, *n* (%)					
No	97 (52.4)	49 (70.0)	88 (49.4)	375 (39.2)	<0.001
Yes	88 (47.6)	21 (30.0)	90 (50.6)	581 (60.8)	
Hemoglobin (mean ± SD, g/L)	10.3(2.3)	10.7 (2.3)	10.4 (2.3)	10.0 (2.4)	0.044
Platelets (mean ± SD, 10^9/L)	187.4 (111.1)	196.0 (90.9)	191. 2(90.1)	197.2 (109.6)	0.667
WBC (mean ± SD, 10^9/L)	15.2 (9.3)	15.5 (12.1)	14.89 (18.0)	15.86 (11.16)	0.751
SOFA (mean ± SD)	4.8 (2.8)	4.2 (2.2)	3.9 (2.2)	3.9 (2.4)	<0.001
Age (mean ± SD)	65.4(15.5)	71.7(16.3)	69.8(15.6)	65.2 (16.6)	<0.001
GCS (mean ± SD, Day 1)	9.8	8.0	13.9	14.6	<0.001
GCS (mean ± SD, Day 2)	11.1	8.2	12.5	14.5	<0.001
GCS (mean ± SD, Day 3)	12.1	8.5	11.9	14.5	<0.001
GCS (mean ± SD, Day 4)	13.6	8.7	11.0	14.5	<0.001
GCS (mean ± SD, Day 5)	14.9	9.0	9.8	14.6	<0.001
ICU stay duration (mean ± SD, day)	11.2 (2.3)	9.5 (3.8)	10.1 (2.4)	10.8 (2.0)	0.291
28-day survive rate (%)	74.1 ± 3.2	65.7 ± 5.7	65.2 ± 3.6	82.4 ± 1.2	<0.001

GCS, Glasgow Coma Scale; WBC, White blood cell; SOFA, SOFA, Sequential Organ Failure Assessment.

### 3.3 28-day survival rates and ICU stay durations of GCS trajectories

**Figure 3:**
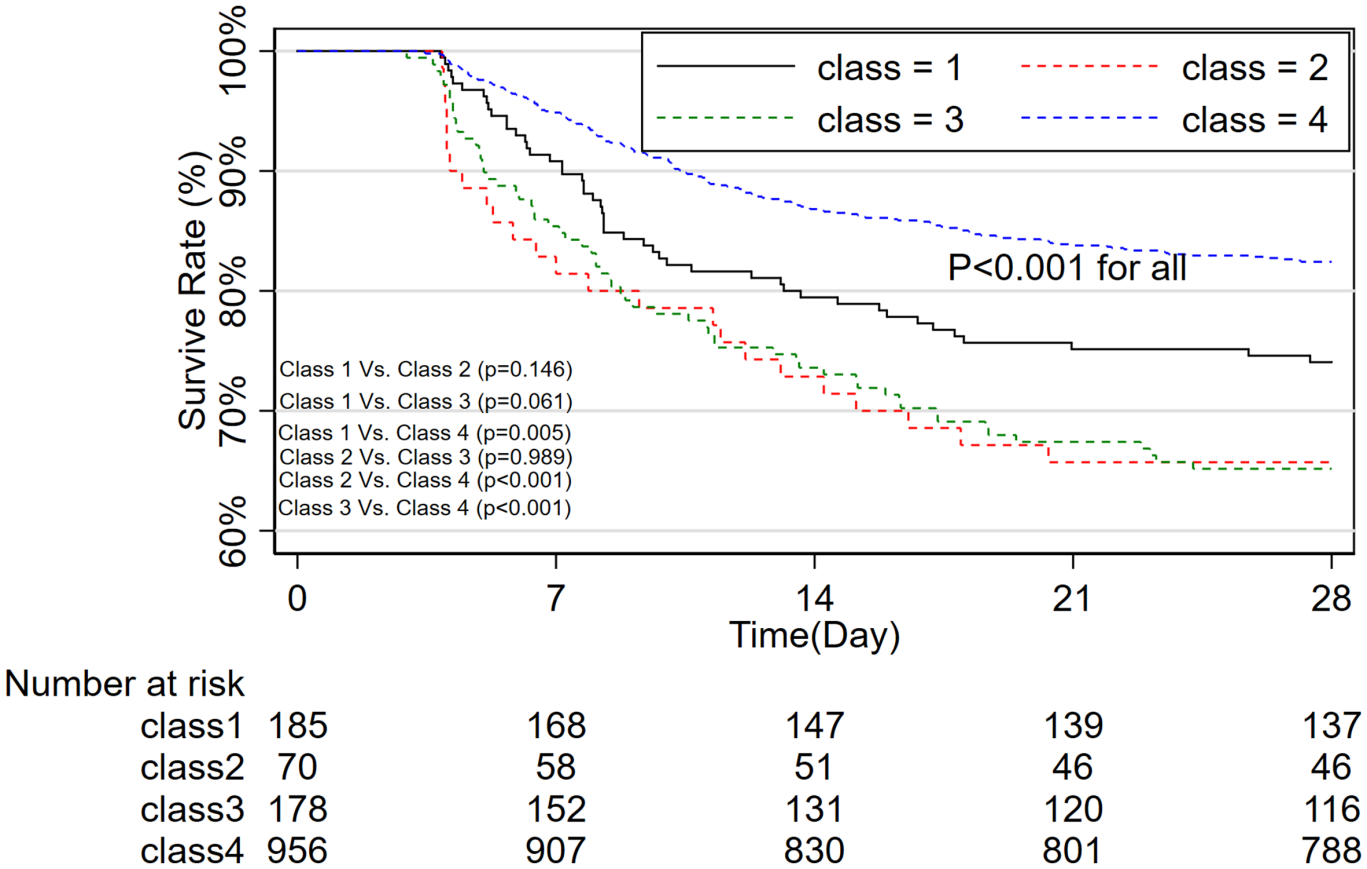
28-Day survival curves and log-rank test for four GCS trajectory groups. GCS, Glasgow Coma Scale.

The primary outcome of the study, 28-day survive rate, revealed significant differences in survival rates among the four GCS trajectory classes. Patients in Class 4, who had persistent high GCS scores, exhibited the highest survival rate of 82.4 ± 1.2%. In contrast, Class 1, the rapid increase group, had a survival rate of 74.1 ± 3.2%. Both Class 2 (slow increase) and Class 3 (rapid decrease) groups had lower survival rates, with Class 2 at 65.7 ± 5.7% and Class 3 at 65.2 ± 3.6%. The differences in survival were statistically significant, with a Log-rank test yielding a *p*-value < 0.001 for all (Figure 3). These results clearly demonstrate that patients in Class 4, who maintained higher and stable GCS levels, had significantly better survival outcomes compared to those whose GCS scores either increased slowly or decreased rapidly. The ICU stay durations for patients in class 1, class 2, class 3, and class 4 were 11.2 ± 2.3 days, 9.5 ± 3.8 days, 10.1 ± 2.4 days, and 10.8 ± 2.0 days, respectively, with no significant statistical difference (*p* = 0.291).

### 3.4 Multivariate cox regression analysis of the four GCS trajectories

To further explore the relationship between GCS trajectories and 28-day survive rate, multivariate Cox regression analyses were performed (Table 3). The crude model, Model1, which considered GCS trajectory classes alone, showed that class 1, class 2, class3 group had significantly increased hazards of death compared to patients in Class 4 (the reference group, all *p* < 0.05). After adjusting for age, gender, and SOFA score in Model 2, the increased risks for class 1, 2, and 3 remained significant (all *p* < 0.05). In Model 3, which adjusted for additional clinical factors including myocardial infarction, chronic pulmonary disease, diabetes, and other comorbidities, the hazard ratios for class 1, class 2 and class 3 remained elevated, suggesting that these GCS trajectory classes were independently associated with a higher risk of mortality. The final model, Model4, which further adjusted for hematological parameters such as hemoglobin, platelet count, and white blood cell count, showed that the hazard ratios for class 1, class 2 and class 3 remained elevated, indicating that other three GCS trajectories was associated with the risk of 28-day all-cause mortality rate.

**Table 3:** Adjusted 28-day survive rate for four GCS trajectory groups after covariate adjustment.

Outcome	Trajectory	Model 1	Model 2	Model 3	Model 4
28-day survive rate	Class 4	Reference	Reference	Reference	Reference
	Class 1	1.58 (1.14–2.17, *p* = 0.005)	1.42 (1.02–1.96, *p* = 0.038)	1.53 (1.10–2.13, *p* = 0.012)	1.52 (1.10–2.14, *p* = 0.012)
	Class 2	1.50 (1.21–1.86, *p* < 0.001)	1.35 (1.09–1.68, *p* = 0.006)	1.38 (1.10–1.73, *p* = 0.005)	1.33 (1.05–1.68, *p* = 0.016)
	Class 3	1.31 (1.19–1.45, *p* < 0.001)	1.27 (1.15–1.40, *p* < 0.001)	1.29 (1.17–1.43, *p* < 0.001)	1.29 (1.16–1.43, *p* < 0.001)

Model 1, the crude model; Model 2, adjusting for age, gender, and SOFA score; Model 3, adjusted for age, gender, SOFA score, myocardial infarction, chronic pulmonary disease, diabetes, and other comorbidities; Model 4, adjusted for age, gender, SOFA score, comorbidities and hematological parameters such as hemoglobin, platelet count, and white blood cell count. GCS, Glasgow Coma Scale.

### 3.5 Subgroup analysis of 28-day survive rate risk for four GCS trajectory groups

**Figure 4:**
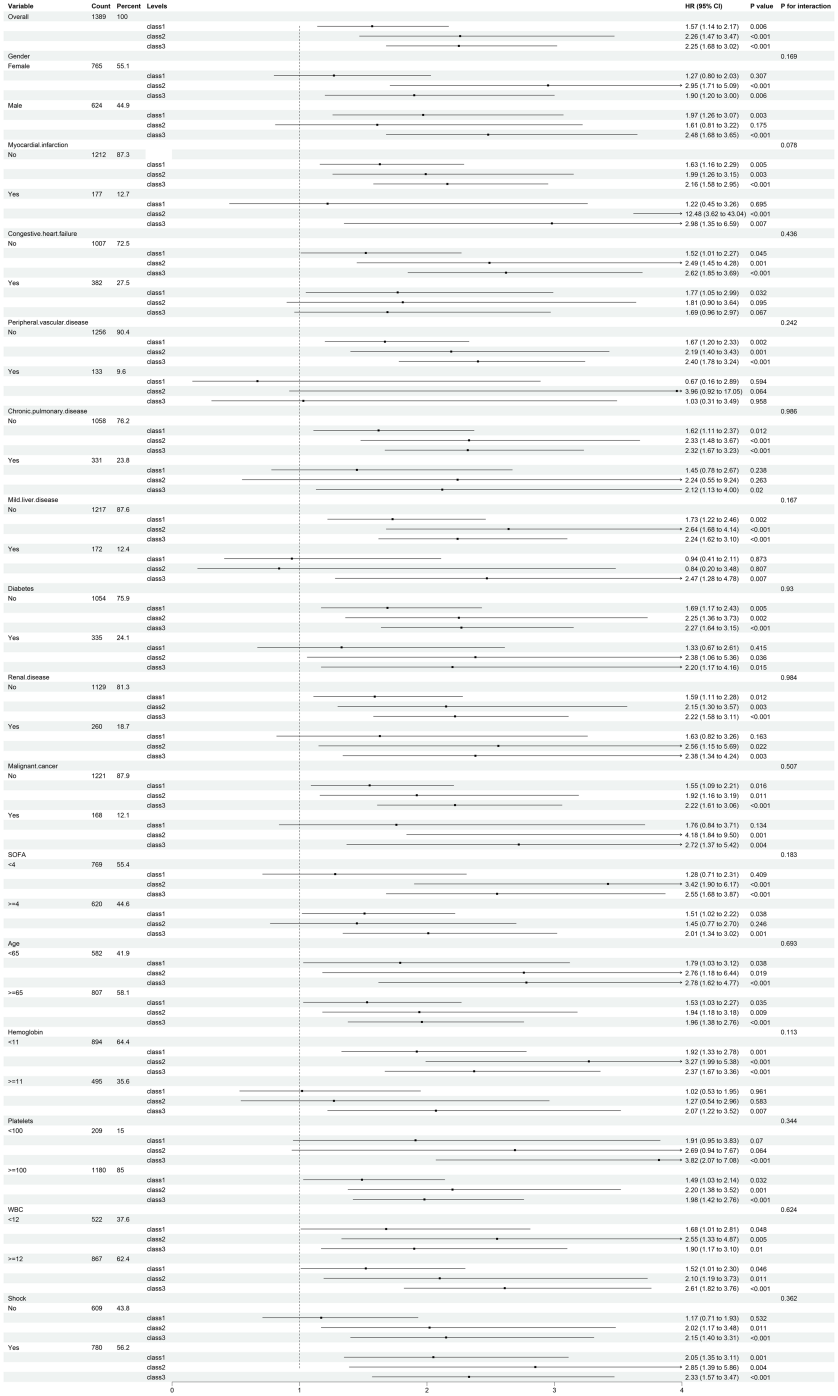
Subgroup analysis of 28-day survive rate risk for four GCS trajectory groups. GCS, Glasgow Coma Scale (The *p*-values refer to the results of the Cox regression method used to compare the risk of 28-day mortality among different subgroups, specifically comparing groups 1, 2, and 3 to trajectory group 4).

Subgroup analyses were conducted to investigate whether the relationship between GCS trajectories and 28-day survive rate varied according to patient characteristics by cox regression analysis Various clinical variables, including age, gender, and the presence of chronic pulmonary disease or shock, were assessed for potential interactions with GCS trajectory classes. Notably, in specific groups of patients without myocardial infarction, peripheral vascular disease, chronic pulmonary disease, mild liver disease, diabetes, renal disease, and malignant cancer, we found significant results. Particularly, when hemoglobin levels were below 11 g/dL and platelet counts were ≥100 × 10^9/L upon ICU admission, the risk of 28-day mortality was markedly increased for patients in classes 1, class 2, and class 3 when compared to those in class 4 (Figure 4). These findings suggest that the impact of dynamic GCS changes on 28-day survive rate is independent of the examined clinical factors and underscores the robustness of the GCS trajectory model as a predictor of patient outcomes. Such results may provide valuable insights for clinical practice, highlighting the importance of monitoring GCS changes and their clinical implications in managing critically ill patients.

## 4 Discussion

The findings of this study significantly contribute to our understanding of the relationship between dynamic changes in GCS scores and 28-day survive rate in patients with SAE. This investigation has not only confirmed prior evidence that GCS scores at the time of ICU admission correlate with outcomes, but it has also illuminated the importance of GCS dynamics preceding ICU admission. The identification of four distinct GCS trajectory classes underscores the heterogeneity of patient responses and provides valuable insights into how patients with septic encephalopathy may evolve over time in their neurological status.

One of the primary implications of this study is that dynamic assessments of cognitive function may offer enhanced prognostic value beyond static GCS scores. Patients assigned to Class 4, who maintained stable and high GCS scores, demonstrated significantly better survival rates 82.4 ± 1.2% compared to those in Class 1 (74.1 ± 3.2%), Class 2 (65.7 ± 5.7%), and Class 3 (65.2 ± 3.6%). This finding suggests that not only the baseline GCS score, but also its trajectory, can influence outcomes. Additionally, the high posterior probabilities for class membership further affirm the validity of our model and enhance its clinical applicability. These observations advocate for incorporating temporal GCS assessments into routine clinical practice, allowing for better prognostic delineation for ICU patients.

The use of trajectory models comes with several advantages and challenges. One of the primary advantages is their ability to capture the heterogeneity of patient responses over time, which is often masked in traditional statistical analyses. This granularity allows for more precise predictions and can lead to improved patient outcomes through tailored interventions (16). Additionally, trajectory models can accommodate missing data, a common issue in longitudinal studies, thereby enhancing their robustness (17). In recent literature, several studies have employed GBTM analysis using data from the MIMIC-IV database to investigate various clinical outcomes in critically ill patients (18, 19). Furthermore, the models can be sensitive to the choice of parameters and the underlying assumptions, which may lead to different conclusions if not carefully validated (20). Addressing these challenges requires ongoing methodological advancements and a deeper understanding of the clinical context in which these models are applied. This approach enhances our understanding of the dynamics of health outcomes, enabling more tailored and effective interventions (21). Furthermore, the study identified specific patient characteristics that correlated with GCS trajectories. For instance, the Class 1 group was younger and less likely to have chronic pulmonary disease or shock, factors known to adversely affect outcomes in critically ill patients. The younger demographic in this group might reflect a better physiological reserve, corresponding with the suggestion that younger patients with less comorbidities demonstrate improved recovery potential compared to their older counterparts. On the other hand, patients in Class 3, characterized by rapid decreases in GCS scores, exhibited high mortality risks due to potential complications arising from their declining neurological status. This disparity across groups signals a need for tailored interventions based on individual trajectories, where patients exhibiting rapid deterioration could benefit from more aggressive monitoring and therapeutic strategies.

The application of multivariate Cox regression analysis further elucidated the independent association of GCS trajectories with mortality risk. Regardless of adjustments for various clinical comorbidities, the relationship remained statistically significant, reinforcing the notion that GCS dynamics can serve as an independent prognostic marker. Notably, patients in Class 3 faced over double the risk of mortality compared to Class 4, reaffirming that a declining GCS is a harbinger of poor outcomes and necessitates immediate clinical attention. These insights suggest that recognizing patterns of change in GCS may help caregivers prioritize interventions in high-risk patients and potentially improve survival outcomes. By focusing on the GCS trajectory, this research provides a more nuanced perspective on how monitoring cognitive function can facilitate early identification of patients at risk for complicated illness courses. Implementing routine cognitive assessments using GCS scores in the ICU could enhance multidisciplinary care approaches, ensuring that neurologically compromised patients receive appropriate interventions, such as optimizing sedation protocols and addressing potential causes of deterioration, including infections, metabolic imbalances, and drug effects.

In subgroup analyses, the impact of the GCS trajectory model on patient prognosis was more pronounced in specific groups of patients without myocardial infarction, peripheral vascular disease, chronic pulmonary disease, mild liver disease, diabetes, renal disease, and malignant cancer. However, we did not observe significant interactions between GCS scores and comorbidities in the interaction tests (p for interaction>0.05). The GCS is a critical tool used in clinical settings to assess a patient’s level of consciousness and neurological function. However, the presence of comorbidities can significantly influence GCS scores, complicating the interpretation of a patient’s neurological status. Comorbidities can introduce additional physiological stressors that may affect brain function and responsiveness. For instance, Studies have shown that patients with comorbidities often experience mental health issues such as anxiety and depression, which can affect their cognitive abilities and consciousness levels. For example, patients with heart failure, due to long-term physical discomfort and psychological stress, may exhibit lower GCS scores (22). This physiological interplay can result in lower GCS scores, which may not solely reflect the severity of the primary neurological insult but also the cumulative burden of these comorbidities. Studies have shown that patients with multiple comorbidities often exhibit poorer outcomes and higher mortality rates, underscoring the importance of considering these factors in the assessment of GCS scores (23, 24). There are significant differences in the impact of different types of comorbidities on GCS scores. Management of comorbidities, such as controlling hypertension and diabetes, can further improve the overall health status of patients (3), thereby enhancing GCS scores. Research has found that patients with chronic pulmonary disease have significantly lower GCS scores during acute exacerbations compared to those without this comorbidity (25–27). A study found that traumatic brain injury patients with comorbid chronic kidney disease had significantly lower GCS scores at discharge compared to those without CKD (28). In addition, GCS scores are also used to assess the impact of other comorbidities on patient prognosis. Studies have shown that patients with serious comorbidities, even with the same GCS score, may have significantly different prognoses (29).

Additionally, the severity of comorbidities is negatively correlated with GCS scores; more severe comorbidities are often associated with lower GCS scores, indicating that the management and treatment of comorbidities are crucial for improving GCS scores. Therefore, clinicians should fully consider the patients’ comorbidities when evaluating GCS to formulate more personalized treatment plans.

Despite the robustness of these findings, certain limitations must be acknowledged. This study is retrospective in nature, meaning that there can be confounding factors not controlled for in the analysis. Residual confounding could remain due to unrecorded variables or variations in clinical practice across different institutions utilizing MIMIC-IV data, especially given the complexity of septic patients. Furthermore, GCS assessments depend on subjective evaluation by clinicians, and variations in inter-rater reliability could introduce bias. Future prospective studies could address these issues, helping to refine the prognostic utility of GCS dynamics in real-world clinical settings.

Future research on the relationship between GCS scores and the prognosis of sepsis-associated encephalopathy should focus on several key areas. First, there is a need for large-scale, multicenter studies that evaluate the long-term cognitive outcomes of patients with varying GCS scores and other prognostic indicators. Such studies could help establish standardized protocols for the assessment and management of SAE, particularly in critical care settings (30). Additionally, exploring the role of novel biomarkers, such as serum neuron-specific enolase (NSE) and glial fibrillary acidic protein (GFAP), in conjunction with GCS scores could provide deeper insights into the pathophysiology of SAE and enhance prognostic accuracy (3). Finally, research should also investigate the effectiveness of targeted therapeutic interventions based on initial GCS assessments to improve outcomes in patients with sepsis-associated encephalopathy. Integrating these approaches may lead to more effective management strategies and better prognostic tools for clinicians treating this complex condition.

In conclusion, this study has demonstrated that dynamic changes in GCS scores leading up to ICU admission are critically associated with 28-day survive rate in patients with septic encephalopathy. The complexity and variability of GCS trajectories underscore the urgent need for ongoing monitoring of neurological status in this patient population. By incorporating dynamic assessments into standard practice, healthcare providers can better stratify risks, tailor interventions, and potentially improve outcomes for patients suffering from this serious complication of sepsis. These findings pave the way for future research endeavors aimed at developing targeted strategies to enhance recovery and diminish mortality in patients with septic encephalopathy.

## Data Availability

The raw data supporting the conclusions of this article will be made available by the authors, without undue reservation.
